# Prognostic value of venous blood analysis at the start of CPR in non-traumatic out-of-hospital cardiac arrest: association with ROSC and the neurological outcome

**DOI:** 10.1186/s13054-020-2762-5

**Published:** 2020-02-22

**Authors:** Ervigio Corral Torres, Alberto Hernández-Tejedor, Rosa Suárez Bustamante, Ramón de Elías Hernández, Isabel Casado Flórez, Antonio San Juan Linares

**Affiliations:** SAMUR-Protección Civil, Madrid, Spain

**Keywords:** Emergency medical services, Out-of-hospital cardiac arrest, Blood gases, Hydrogen-ion concentration

## Abstract

**Background:**

The knowledge of new prognostic factors in out-of-hospital cardiac arrest (OHCA) that can be evaluated since the beginning of cardiopulmonary resuscitation (CPR) manoeuvres could be helpful in the decision-making process of prehospital care. We aim to identify metabolic variables at the start of advanced CPR at the scene that may be associated with two main outcomes of CPR (recovery of spontaneous circulation (ROSC) and neurological outcome).

**Methods:**

Prospective observational study of all non-traumatic OHCA in patients older than 17 years assisted by emergency medical services (EMS), with doctor and nurse on board, between January 2012 and December 2017. Venous blood gases were sampled upon initially obtaining venous access to determine the initial values of pH, pCO_2_, HCO_3_^−^, base excess (BE), Na^+^, K^+^, Ca^2+^ and lactate. ROSC upon arrival at the hospital and neurological status 30 days later (Cerebral Performance Categories (CPC) scale) were recorded.

**Results:**

We included 1552 patients with OHCA with blood test data in a 6-year period. ROSC was achieved in 906 cases (58.4%), and good neurological recovery at 30 days (CPC I-II) occurred in 383 cases (24.68%). In multivariate analysis, we found a significant relationship between non-recovery of spontaneous circulation (no-ROSC) and low pH levels (adjusted odds ratio (OR) 0.03 (0.002–0.59), *p* = 0.020), high pCO_2_ levels (adjusted OR 1.03 [1.01–1.05], *p* = 0.008) and high potassium levels (adjusted OR 2.28 [1.43–3.61], *p* = 0.008). Poor neurological outcomes were associated with low pH levels (adjusted OR 0.06 [0.02–0.18], *p* < 0.001), high pCO_2_ (adjusted OR 1.05 [1.03–1.08], *p* < 0.001), low HCO_3_^−^ (adjusted OR 0.97 [0.94–0.999], *p* = 0.044), low BE (adjusted OR 0.96 [0.93–0.98], *p* < 0.001) and high potassium levels (adjusted OR 1.37 [1.16–1.60], *p* < 0.001).

**Conclusion:**

There is a significant relationship between severe alterations of venous blood-gas variables and potassium at the start of CPR of non-traumatic OHCA and low-ROSC rate and neurological prognosis.

## Background

Medical attention to out-of-hospital cardiac arrest (OHCA) entails a variety of special challenges, besides the inherent ones when coping with this type of pathology, in any context. One of them would be the lack of exactness when estimating the time of the cardiac arrest (CA), which may be of utter relevance. No-flow time is probably one of the key factors in the neurological outcome [1–3]. Some clinical signs have been considered to estimate the cardiac arrest (CA) onset time but they are not reliable enough to influence on the decision to initiate, continue or stop the cardiopulmonary resuscitation (CPR).

Along with the cardiac arrest onset and first monitored rhythm, it seems that the metabolic status during CPR would be another important factor with a relevant impact on the probability of survival and on the neurological outcome [4].

Multiple responses have been sought in this regard, analysing factors that could be related to the neurological outcome and, therefore, that could lead to determine whether to maintain or to stop all resuscitation efforts. In most cases, such studies analyse the patient’s status after the recovery of spontaneous circulation (ROSC), either on arrival at the emergency department or at the critical-care unit in the hospital. Thus, the interaction between the forementioned neurological outcome and certain aspects of the CA has been evaluated, [5–9] such as the first monitored rhythm, the presence of first responders or the duration of resuscitation efforts, in some cases using scales and other predictive tools [10, 11].

Several blood metabolite concentrations were evaluated while looking for possible predictive tools and obtaining positive results. Recently, an important contribution was made in this search for predictors to associate blood metabolites and the neurological outcome [12]. Amongst them, the relationship of variables, such as PaCO_2_ or lactate, and the chance of survival with a good neurological outcome are remarkable. Several studies have demonstrated the statistical association between hypocapnia [13, 14] and neurological damage. Likewise, high levels of lactate [15, 16] at 6 and 12 h after resuscitation can be significant predictors of poor neurological outcome, but it does not seem to operate in earlier stages [17]. The relationship with pH and other blood-gas values has also been assessed but almost always after resuscitation [18–20]. Measurements after the transference to the hospital can be accurate predictors but are not suitable for the emergency medical service (EMS) team and are influenced by advanced CPR.

In Spain and some other countries where doctors and nurses are also working in mobile advanced life support (ALS) units, the common course of action is to do CPR on the field and transfer the patient to the hospital if ROSC is achieved or declare him/her dead at the scene in the opposite case. Only in very specific situations, such as patients who recover and lose spontaneous circulation several times, or if any other mechanical alternative is considered feasible, may the patient be transferred with ongoing CPR.

This study aims at analysing the metabolic situation at the start of advanced CPR at the scene and its relation with the neurological condition after 1 month. Our group has wide experience with the use of point-of-care analysis on the field. Since there is often very limited data regarding a patient’s previous medical history and time of arrest, on-scene lab studies (at the beginning of CPR) could be useful for decision-making and provide some kind of metabolic watch of the patient and therefore become a tool to determine the likelihood of successful resuscitation [21, 22].

We aim to study the association between the analytical variables at the start of advanced CPR and two main outcomes: ROSC at arrival to the hospital and the neurological condition of the patients 30 days after the event, assessed by the Cerebral Performance Categories (CPC) scale, considering intact neurological recovery grades I and II.

## Methods

### Study design and setting

This was a prospective observational cohort study of all non-traumatic OHCA in patients older than 17 years assisted between 2012 and 2017 by the EMS SAMUR-PC. EMS participants collected the data at the scene following the Utstein style. Epidemiological variables were recorded (age, sex, rhythm of cardiac arrest onset, witnessed cardiac arrest, previous manoeuvres by first responders/bystanders).

As stated in the procedures of this EMS, [23] in every attended cardiac arrest, a venous blood sample was taken upon initially obtaining venous access (in the first 90 s) and analysed on the scene through the point-of-care EPOC® (Epocal Inc., Ottawa, Canada) device. This venous access was almost always achieved in the arms. If it was not possible, intraosseous access was achieved. These initial blood test results (pH, pCO_2_, HCO_3_^−^, base excess [BE], Na^+^, K^+^, Ca^2+^ and lactate), prior to the administration of any drug, were analysed as independent variables in this study.

Cases in which CPR was not initiated, the patient was under aged or blood analysis could not be performed were excluded.

SAMUR-PC is an EMS whose scope of action is developed in all public settings of the city of Madrid (Spain), with a population of 3.2 million inhabitants and 140,000 dispatches per year. A physician and a nurse are on board all mobile ALS units. In every suspected very severe patient, a mobile ALS unit is dispatched, as well as the chief doctor and the head nurse.

Our service is enrolled in an international study on ECPR. The four cases included in that study have not been considered for the present one. When ROSC is not achieved after efforts according to recommendations—and the patient would otherwise be declared dead—he/she could be transferred to the hospital in asystole if meeting the inclusion criteria for uncontrolled non-heart beating donation. In the present study, these patients are included in the no-ROSC group.

### Study endpoints

The dependent variables were ROSC upon arrival at the hospital and intact neurological survival (CPC I-II) at 30 days. CPC scale was performed by in-person interview by the responsible physician of the patient at the hospital.

### Statistical analysis

The description of quantitative variables was performed with central tendency and dispersion indices based on rankings (mean ± standard deviation). The description of categorical variables was performed with absolute and relative frequencies in percentages.

Proportions were compared with Pearson’s chi-squared tests to analyse the relationships between categorical variables. Quantitative variables were analysed with the Student’s *t* test.

In the first instance, a univariate analysis was carried out using simple binary logistic regression between each independent variable and the dependent variables.

To assess the relationship of the independent variables associated with the dependent variables, we used multivariate binary logistic regression models, one for each of the variables (pH, pCO_2_, HCO_3_^−^, BE, Na^+^, K^+^, Ca^2+^ and lactate). Each model was evaluated separately for each independent variable of interest and was adjusted with the following covariables, selected based on their relationship with the dependent variables in the scientific literature [3]: age, sex, first monitored rhythm, witnessed CA, previous manoeuvres and the interactions of these covariates with each independent variable of interest. Estimative, multivariate, binary, logistic regressions were performed, selecting the final model by using the backward procedure with the criterion of statistical significance to remove covariates. The likelihood ratio test was used to analyse the overall statistical significance of the models. The Wald test was used for the individual statistical significance of the predictors. The magnitude of the effect of each independent variable was expressed with the odds ratio (OR) and its 95% confidence interval (CI). In continuous variables, it was analysed using intervals of increment or decrement and OR was expressed per unit of analyte. Internal validation of the final multivariate models was carried out by dividing the sample by time criteria, using the most recent 80% of the sample to estimate the models and the oldest 20% of the sample to validate the models. Values of *p* < 0.05 were considered significant. The statistical treatment was performed with the SPSS, version 18 (SPSS Inc., Chicago, IL, USA) statistical package.

## Results

**Fig. 1:**
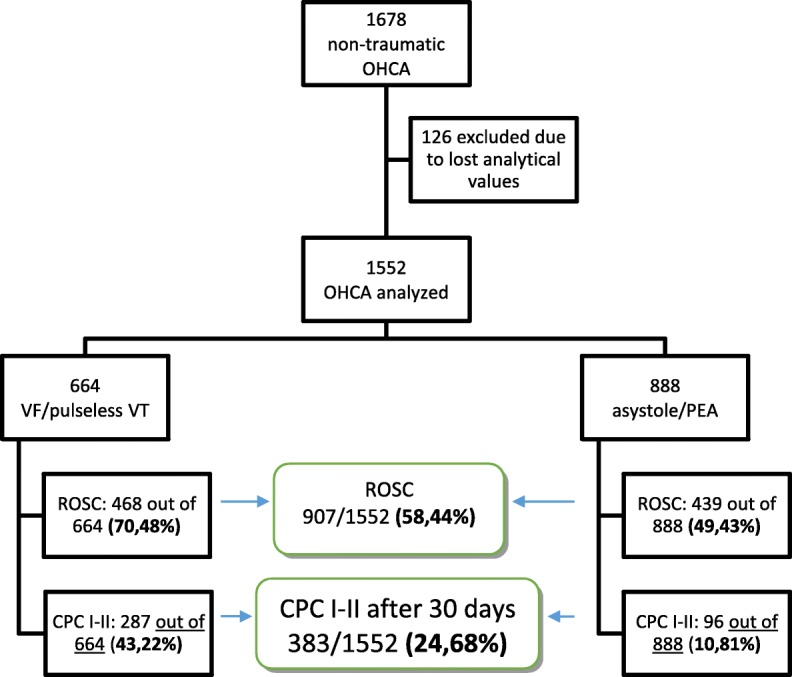
Study scenario. Central green boxes summarise data from all the records included. CPC I-II percentages are out of all patients, not out of patients who recovered spontaneous circulation

A total of 1678 OHCAs were consecutively attended by the EMS. One hundred and twenty-six cases (7.5%) were lost due to not being able to obtain the blood analytical data due to an ambient temperature that made the device unusable. Thus, the study was performed on 1552 records (Fig. 1). Only 3% of them occurred at home/residence (the rest of them were in the street or public buildings, including workplace, gyms and educational institutions) and 91% were witnessed. The mean response time (from incoming call to arrival of the first vehicle) was 8 min and 14 s.

Epidemiological characteristics of the population studied are shown in Table 1. In 50.2% of cases, first responders/bystanders performed CPR prior to EMS arrival. ROSC was achieved in 907 patients (58.44%) patients; in the subgroup of shockable rhythms, ROSC was achieved in 70.4% of cases. Table 2 shows the association of ROSC and good neurological recovery with other epidemiological variables. In 24.68% of 1552 patients, there was no neurological damage or only minimal sequelae after 1 month (CPC I-II); in the subgroup of shockable rhythms, this percentage was 43.2%.

**Table 1:** Epidemiological values of the study population (*n* = 1552)

Condition		*n* (%)
Gender	Male	1234 (79.5)
	Female	318 (20.5)
Age (years)	Global	64.83 ± 15.62
	Male	63.56 ± 15.19
	Female	69.76 ± 16.31
First monitored rhythm	VF/pulseless VT	664 (42.78)
	Asystole	741 (47.75)
	PEA	147 (9.47)
Previous CPR by witnesses	No previous CPR	599 (38.60)
	Untrained personnel	469 (30.22)
	Trained personnel	310 (19.97)
	SAMUR-PC staff	174 (11.21)

*CPR* cardiopulmonary resuscitation, *PEA* pulseless electrical activity, *VF* ventricular fibrillation, *VT* ventricular tachycardia

Data are expressed as mean values ± standard deviation for continuous variables and number of cases (percentage) for categorical variables

**Table 2:** Association of ROSC (*n* = 907) and good neurological recovery CPC I-II (*n* = 383) with other epidemiological variables

	Condition		*n* (%) of them who got ROSC	*p*
ROSC	Gender	Male	717/1234 (58.10)	0.596
		Female	190/318 (59.75)	
	First monitored rhythm	VF/pulseless VT	468/664 (70.48)	< 0.001
		Asystole/PEA	439/888 (49.43)	
	Previous CPR by witnesses	No previous CPR	327/599 (54.59)	0.01
		Untrained personnel	275/469 (58.64)	
		Trained personnel	181/310 (58.39)	
		SAMUR-PC staff	124/174 (71.26)	
	Condition		*n* (%) of them who got CPC I-II	*p*
CPC I-II	Gender	Male	333/1234 (26.99)	< 0.001
		Woman	50/318 (15.72)	
	First monitored rhythm	VF/pulseless VT	287/664 (43.22)	< 0.001
		Asystole/PEA	96/888 (10.81)	
	Previous CPR by witnesses	No previous CPR	112/599 (18.70)	< 0.001
		Untrained personnel	118/469 (25.16)	
		Trained personnel	79/310 (25.48)	
		SAMUR-PC staff	74/174 (42.53)	

*CPR* cardiopulmonary resuscitation, *PEA* pulseless electrical activity, *ROSC* recovery of spontaneous circulation, *VF* ventricular fibrillation, *VT* ventricular tachycardia

Data are expressed as mean values ± standard deviation for continuous variables and number of cases (percentage) for categorical variables

In the univariate analysis, a significant relationship was found between non-recovery of spontaneous circulation (no-ROSC) and lower pH and higher levels of pCO_2_, potassium and calcium (Table 3). Likewise, there was a significant relationship between poor neurological recovery (no-CPC I-II) and a decrease in pH, HCO_3_^−^ and BE and an increase in pCO_2_ and potassium (Table 3).

**Table 3:** Comparison of analytical values according to ROSC and neurological recovery CPC I-II (univariate analysis)

ROSC		Patients with ROSC	Patients without ROSC	OR (CI95%)	*p*
	pH	7.129 ± 0.167	7.109 ± 0.177	0.508 (0.282–0.914)	0.024
	pCO_2_ (mmHg)	69.92 ± 26.16	73.70 ± 28.48	1.005 (1.001–1.009)	0.007
	HCO_3_^−^ (mmol/l)	22.55 ± 4.73	22.31 ± 4.84	0.990 (0.969–1.011)	0.328
	BE (mmol/l)	− 6.66 ± 7.12	− 7.35 ± 7.44	0.988 (0.974–1.002)	0.089
	Lactate (mmol/l)	7.23 ± 11.72	7.21 ± 8.42	1.00 (0.990–1.010)	0.964
	K^+^ (mmol/l)	4.22 ± 1.01	4.55 ± 1.28	1.293 (1.180–1.416)	0.001
	Na^+^ (mmol/l)	138.53 ± 12.97	139.57 ± 8.04	1.010 (0.999–1.021)	0.083
	Ca^++^ (mmol/l)	1.34 ± 4.48	1.17 ± 0.21	0.580 (0.347–0.971)	0.038
CPC I-II		Patients with CPC I-II	Patients without CPC I-II	OR (CI95%)	*p*
	pH	7.196 ± 0.147	7.096 ± 0.172	0.017 (0.08–0.040)	< 0.001
	pCO_2_ (mmHg)	60.72 ± 22.42	75.02 ± 27.71	1.027 (1.021–1.033)	< 0.001
	HCO_3_^−^ (mmol/l)	22.97 ± 4.19	22.29 ± 4.94	0.970 (0.947–0.994)	0.015
	BE (mmol/l)	− 5.15 ± 6.08	− 7.54 ± 7.52	0.948 (0.930–0.966)	< 0.001
	Lactate (mmol/l)	7.11 ± 17.63	7.26 ± 6.63	1.002 (0.989–1.014)	0.804
	K^+^ (mmol/l)	3.95 ± 0.79	4.49 ± 1.21	1.725 (1.506–1.977)	< 0.001
	Na^+^ (mmol/l)	139.20 ± 12.67	138.88 ± 10.68	0.997 (0.986–1.008)	0.620
	Ca^++^ (mmol/l)	1.55 ± 6.88	1.18 ± 0.23	0.712 (0.431–1.175)	0.183

*BE* base excess, *CI* confidence interval, *OR* odds ratio (per unit of analyte in all of them except for pH where it is per tenth of unit), *ROSC* recovery of spontaneous circulation

Data are expressed as mean values ± standard deviation

A significant relationship between non-recovery of spontaneous circulation (no-ROSC) and low pH and high pCO_2_ and potassium levels was found in the multivariate analysis using intervals of increment or decrement. The internal validation of the models was positive for pH and pCO_2_ and negative for potassium (Table 4). In the same way, the multivariate analysis showed a significant relationship between neurological non-recovery (no-CPC I-II) and low pH, HCO_3_^−^ and BE and high pCO_2_ and potassium levels. The internal validation of the models was positive for pH, pCO_2_, BE and potassium and negative for HCO_3_^−^ (Table 4).

**Table 4:** Analysis with multivariate binary logistic regression for ROSC and good neurological recovery (CPC I-II)

	OR (CI 95%)	*p*	*R*^2^ by Nagelkerke	Validation of the model
ROSC				
pH	0.034 (0.002–0.587)	0.020	0.096	Yes
pCO_2_ (mmHg)	1.028 (1.007–1.050)	0.008	0.092	Yes
K^+^ (mmol/l)	2.275 (1.434–3.609)	< 0.001	0.106	Do not
Ca^2+^ (mmol/l)	0.717 (0.407–1.260)	0.247	0.091	Do not
Good neurological outcome (CPC I-II)				
pH	0.06 (0.02–0.18)	< 0.001	0.28	Yes
pCO_2_ (mmHg)	1.05 (1.03–1.08)	< 0.001	0.28	Yes
HCO_3_^−^ (mmol/l)	0.97 (0.94–0.999)	0.044	0.26	Do not
BE (mmol/l)	0.96 (0.93–0. 98)	< 0.001	0.27	Yes
K^+^ (mmol/l)	1.37 (1.16–1.6)	< 0.001	0.27	Yes

*BE* base excess, *CI* confidence interval, *OR* odds ratio (per unit of analyte)

**Fig. 2:**
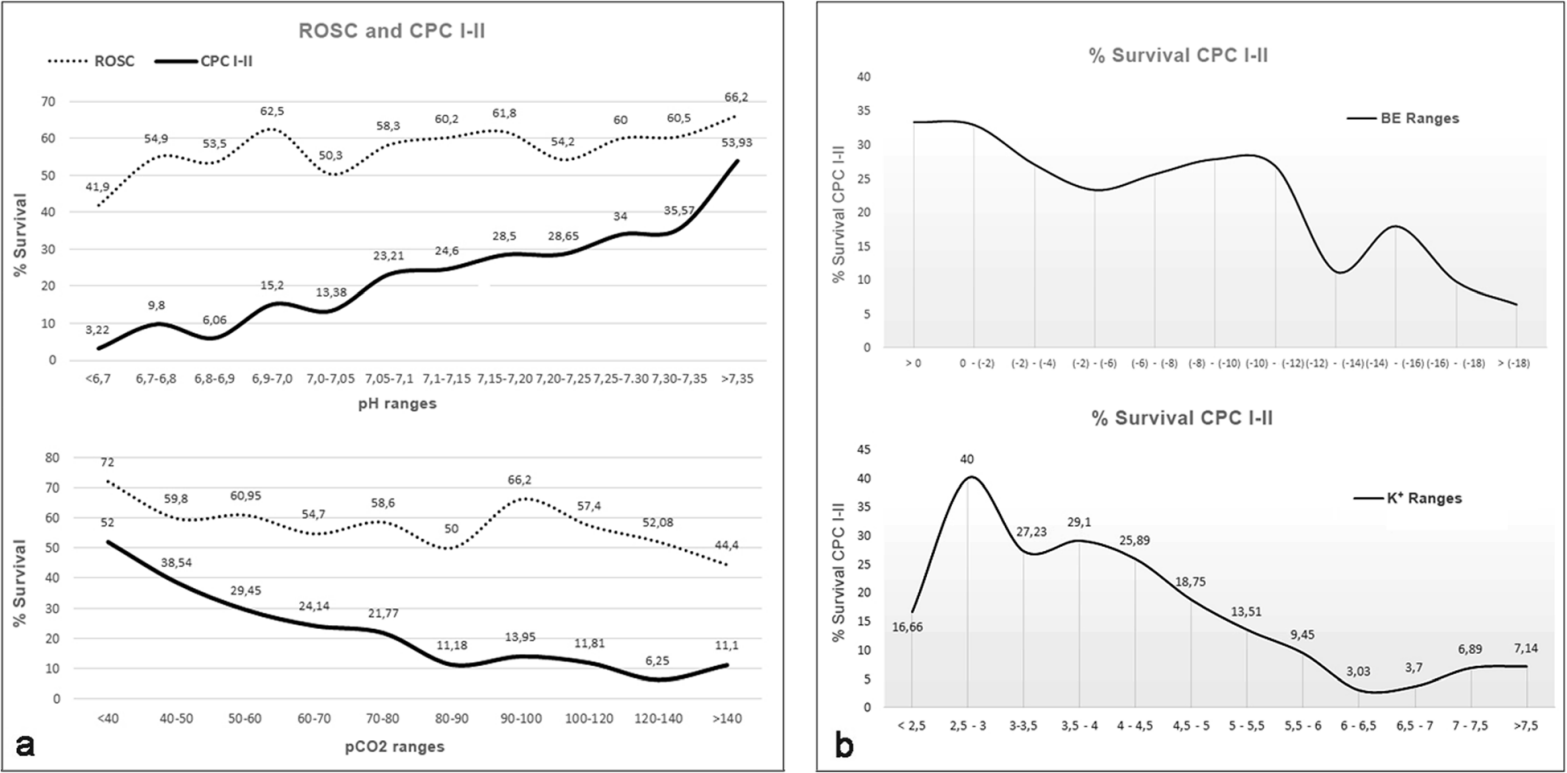
**a** Relationship between the percentages of recovery of spontaneous circulation (ROSC) and 30-day survival with good neurological outcome (CPC I-II) and the values of pH and pCO_2_ expressed in ranges evaluated in the multivariate binary logistic regression. **b** Relationship between the percentages of survival with good neurological outcome (CPC I-II) and the values of base excess (BE) and K^+^ expressed in ranges evaluated in the multivariate binary logistic regression. Venous blood-gas variables, including alterations in blood potassium, are associated with neurological outcomes. Low pH, a raised pCO_2_ and a high base deficit, as well as either very low or high blood concentration of potassium, were associated with worse outcome

We continuously assessed the relationship between the values of the different analytes and the outcome variables (Fig. 2), after noticing a proportional relationship between the levels of each predictor and those variables. This relationship was very close when referring to the values of pH and pCO_2_, both for ROSC and for good neurological recovery.

## Discussion

The importance of these analyses lies in the fact that the study was carried out in a setting in which all patients were attended by emergency teams with physician, nurse and several emergency technicians (paramedics), usually helped by an additional chief physician and/or nurse, who performed all the ALS manoeuvres on the scene either until the recovery of the pulse or until certified death; with very few exceptions, patients were only taken to hospital if ROSC was achieved. These characteristics, in addition to others already mentioned, differentiate this study from others published previously [4], in which ALS techniques were performed only in the hospital after being transferred, maybe having received only basic life support manoeuvres, which implies a prolongation of the time of low cerebral flow, which can affect the final results.

In our study, the analysis was performed on scene and makes it possible to have blood results even in cases of patients declared dead on scene, which was not possible in other studies analysing blood sample upon arrival at the hospital.

Thus, according to these findings, the metabolic data collected at the arrival of the team, at the beginning of ALS on scene through a venous sample [24]—undervalued in the current international guidelines [25, 26]—should be considered as a factor to be taken into account in cardiac arrest management in the out-of-hospital setting. The goal would not be just estimating the duration of the arrest and/or the quality of bystander CPR, but trying to estimate the probability of ROSC and neurological outcome, even in patients with prolonged CPR.

An interesting study with some similarities with ours revealed the association between blood variables such as pH and K^+^ and the prognosis of the patient [4]. However, most patients included in that study received ALS manoeuvres once in the hospital, and some confounding factors were not considered in the statistical analysis, such as the initial rhythm, presence of first respondents or the age of the patients.

The relationship between the analytical values and the clinical outcomes is remarkable, as can be seen from the magnitude of the effect of these variables: the risks of no-ROSC and no-neurological recovery increase, on average, for each unit of increase in pCO_2_ (mmHg), 5% and 3%, respectively. In the same way, the risks of no-ROSC and no-neurological recovery decrease, on average, for each tenth of increase in pH, by 9.4% and 9.7%, respectively. Similar values can be deduced from Table 4.

These results are coincident, in terms of the influence on these two clinical outcomes (ROSC and neurological outcome CPC I-II), with those of some of the epidemiological factors assessed in this work, such as the first monitored rhythm, the presence of first responders, the age or sex, although in the last two we only managed to establish an association with neurological recovery (Table 2).

Lactate values were analysed in previous studies for its potential prognostic value in critical-care units, with positive results as a prognostic factor to predict the patient’s progress when measured after ROSC [27]. However, in our study, with lactate measurement at the start of resuscitation, we did not observe a significant statistical relationship with ROSC or with neurological recovery. This was surprising to us. Interestingly, base excess was associated with outcomes. This may be due to the presence of other metabolites or not measured weak acids.

Figure 2 shows the parallelism between the percentages of ROSC and neurological recovery with values of pH and pCO_2_ closer to normal. In our study, no survival was obtained with an optimal neurological recovery in patients with a pH lower than 6.60. Of particular interest was the bimodal graph that relates kalemia with CPC I-II survival, where both the levels of hypokalemia and hyperkalemia (mainly the latter) were related to survival with worse neurological outcome, as was stated by Lin et al. [28].

Our study has some limitations. First, it was a study carried out in a single geographical area of action with specific assistance. Our good results could be explained by several factors: (1) Our EMS is based on physicians and nurses on board and in all severe cases the chief doctor and the head nurse are commissioned to collaborate with the team. (2) The scope of our service is the public area of a big city; only a scarce number of cases occur in homes, so it may probably influence the profile of patients. (3) Our response times are lower than those in other studies. (4) Training is of great importance in our institution, with frequent mandatory courses and sessions. (5) There is fluid feedback from hospitals to improve our quality standards. (6) SAMUR-PC has, as one of its main objectives, the citizenship education. The first responder programme is aimed mainly at members of security forces and firefighters.

Second, 126 cases were lost because a partial or full blood analysis could not be performed due to high or low ambient temperatures that limited the use of the EPOC® device, given that the analyser needs to be in a certain interval to be operative and reliable. The possible relationship of those extreme temperatures with the cause of the CA and the probability of recovery is unknown. The percentage of these cases is under 10% and would probably have a minor effect on statistical analysis. Anyway, it could be speculated that colder ambient conditions could improve the neurological prognosis as well as the opposite. Third, blood samples were obtained when the first venous (or intraosseous) line was achieved, but the time from the start of CPR to that achievement was not recorded. It is known that the local metabolic condition (in the limb of the venous access) may not be exactly the same of that in the rest of the body, but it is the one we can analyse and it is the best available surrogate. Previous studies used the same method. Fourth, the cause of cardiac arrest and the no-flow/low-flow time are unknown. Some aetiologies could be related to a worse initial metabolic state if the patient was already deteriorating before cardiac arrest.

Our approach is limited when trying to identify cases where CPR would be futile. This would require a different approach. It is really unlikely that we can find a threshold, except strikingly extreme values, above or below which continued resuscitation is surely futile. However, knowing which biomarkers predict outcome could be helpful when deciding to maintain a prolonged CPR effort. This could be challenging for future directions in investigation.

Finally, we have assumed monotonic relationships between continuous variables and outcomes. This is probably true for most of them but potassium levels will probably have a more complex relationship with outcome and this may underestimate the association.

Although this study was designed to find variables related with ROSC and CPC I-II survival, we feel that the importance of blood analysis on scene goes further, i.e. identifying the severity of this or other conditions so further research could follow these directions.

## Conclusions

In the context of the non-traumatic OHCA, our data show the association between gas test abnormalities at the start of CPR and worse clinical outcome in terms of lower ROSC rate and higher CPC scale grade. Given the difficulties obtaining reliable data in this area, these metabolic variables at the beginning of CPR are of great diagnostic and prognostic value. Further studies should address the usefulness of these measurements at the start of resuscitation for decision-making.

## Data Availability

The datasets used and/or analysed during the current study are available from the corresponding author on reasonable request.

## Abbreviations

ALS: Advanced life support
BE: Base excess
CA: Cardiac arrest
CI: Confidence interval
CPC: Cerebral Performance Categories
CPR: Cardiopulmonary resuscitation
EMS: Emergency medical service
OHCA: Out-of-hospital cardiac arrest
OR: Odds ratio
ROSC: Recovery of spontaneous circulation

## References

[1] Sasson C, Rogers MA, Dahl J, Kellermann AL (2010). Predictors of survival from out-of-hospital cardiac arrest: a systematic review and meta-analysis. Circ Cardiovasc Qual Outcomes.

[2] Travers AH, Perkins GD, Berg RA, Castren M, Considine J, Escalante R (2015). Basic life support chapter collaborators. Part 3: adult basic life support and automated external defibrillation: 2015 International Consensus on Cardiopulmonary Resuscitation and Emergency Cardiovascular Care Science With Treatment Recommendations. Circulation.

[3] Adnet F, Triba MN, Borron SW, Lapostolle F, Hubert H, Gueugniaud PY (2017). Cardiopulmonary resuscitation duration and survival in out-of-hospital cardiac arrest patients. Resuscitation..

[4] Shin J, Lim YS, Kim K, Lee HJ, Lee SJ, Jung E (2017). Initial blood pH during cardiopulmonary resuscitation in out-of-hospital cardiac arrest patients: a multicenter observational registry-based study. Crit Care.

[5] Nolan JP, Neumar RW, Adrie C, Aibiki M, Berg RA, Böttiger BW (2008). Post-cardiac arrest syndrome: epidemiology, pathophysiology, treatment, and prognostication. A scientific statement from the International Liaison Committee on Resuscitation; the American Heart Association Emergency Cardiovascular Care Committee; the Council on Cardiovascular Surgery and Anesthesia; the Council on Cardiopulmonary, Perioperative, and Critical Care; the Council on Clinical Cardiology; the Council on Stroke. Resuscitation.

[6] Sandroni C, Cavallaro F, Callaway CW, Sanna T, D'Arrigo S, Kuiper M (2013). Predictors of poor neurological outcome in adult comatose survivors of cardiac arrest: a systematic review and meta-analysis. Part 1: patients not treated with therapeutic hypothermia. Resuscitation..

[7] Sandroni C, Cavallaro F, Callaway CW, Sanna T, D'Arrigo S, Kuiper M (2013). Predictors of poor neurological outcome in adult comatose survivors of cardiac arrest: a systematic review and meta-analysis. Part 2: patients treated with therapeutic hypothermia. Resuscitation..

[8] Kaji AH, Hanif AM, Bosson N, Ostermayer D, Niemann JT (2014). Predictors of neurologic outcome in patients resuscitated from out-of-hospital cardiac arrest using classification and regression tree analysis. Am J Cardiol.

[9] Lin YN, Chang SS, Wang LM, Chi HT, Ueng KC, Tsai CF (2017). Prehospital predictors of initial shockable rhythm in out-of-hospital cardiac arrest: findings from the Taichung Sudden Unexpected Death Registry (THUNDER). Mayo Clin Proc.

[10] Aschauer S, Dorffner G, Sterz F, Erdogmus A, Laggner A (2014). A prediction tool for initial out-of-hospital cardiac arrest survivors. Resuscitation..

[11] Skrifvars MB, Varghese B, Parr MJ (2012). Survival and outcome prediction using the apache III and the out-of-hospital cardiac arrest (OHCA) score in patients treated in the intensive care unit (ICU) following out-of-hospital, in-hospital or ICU cardiac arrest. Resuscitation..

[12] Huntgeburth M, Adler C, Rosenkranz S, Zobel C, Haupt WF, Dohmen C (2014). Changes in neuron-specific enolase are more suitable than its absolute serum levels for the prediction of neurologic outcome in hypothermia-treated patients with out-of-hospital cardiac arrest. Neurocrit Care.

[13] Schneider AG, Eastwood GM, Bellomo R, Bailey M, Lipcsey M, Pilcher D (2013). Arterial carbon dioxide tension and outcome in patients admitted to the intensive care unit after cardiac arrest. Resuscitation..

[14] Lee BK, Jeung KW, Lee HY, Lee SJ, Jung YH, Lee WK (2014). Association between mean arterial blood gas tension and outcome in cardiac arrest patients treated with therapeutic hypothermia. Am J Emerg Med.

[15] Cocchi MN, Miller J, Hunziker S, Carney E, Salciccioli J, Farris S (2011). The association of lactate and vasopressor need for mortality prediction in survivors of cardiac arrest. Minerva Anestesiol.

[16] Starodub R, Abella BS, Grossestreuer AV, Shofer FS, Perman SM, Leary M (2013). Association of serum lactate and survival outcomes in patients undergoing therapeutic hypothermia after cardiac arrest. Resuscitation..

[17] Sarıaydın T, Çorbacıoğlu ŞK, Çevik Y, Emektar E (2017). Effect of initial lactate level on short-term survival in patients with out-of-hospital cardiac arrest. Turk J Emerg Med.

[18] Ganga HV, Kallur KR, Patel NB, Sawyer KN, Gowd PB, Nair SU (2013). The impact of severe acidemia on neurologic outcome of cardiac arrest survivors undergoing therapeutic hypothermia. Resuscitation..

[19] Vaahersalo J, Bendel S, Reinikainen M, Kurola J, Tiainen M, Raj R (2014). Arterial blood gas tensions after resuscitation from out-of-hospital cardiac arrest: associations with long-term neurologic outcome. Crit Care Med.

[20] Momiyama Y, Yamada W, Miyata K, Miura K, Fukuda T, Fuse J (2017). Prognostic values of blood pH and lactate levels in patients resuscitated from out-of-hospital cardiac arrest. Acute Med Surg.

[21] Corral E, Casado MI, García-Ochoa MJ, Suárez R. Looking at “metabolic watch”. The analytical parameters found at the beginning of the resuscitation are predictors of the neurological prognostic in the prehospital cardiac arrest. Resuscitation. 2015;96 Suppl:148.

[22] Hernández-Tejedor A, Corral E, de Elías R, Suárez R (2019). CPR by first responders improves acid-base balance and prognosis in out-of-hospital non-traumatic cardiac arrest. BMJ Open.

[23] SAMUR-Protección Civil (2019). Procedures manual.

[24] Zeserson E, Goodgame B, Hess JD, Schultz K, Hoon C, Lamb K (2018). Correlation of venous blood gas and pulse oximetry with arterial blood gas in the undifferentiated critically ill patient. J Intensive Care Med.

[25] Soar J, Nolan JP, Böttiger BW, Perkins GD, Lott C, Carli P (2015). Adult advanced life support section collaborators. European Resuscitation Council guidelines for resuscitation 2015: section 3. Adult advanced life support. Resuscitation.

[26] Mancini ME, Diekema DS, Hoadley TA, Kadlec KD, Leveille MH, McGowan JE (2015). Part 3: ethical issues: 2015 American Heart Association guidelines update for cardiopulmonary resuscitation and emergency cardiovascular care. Circulation..

[27] Donnino MW, Miller J, Goyal N, Loomba M, Sankey SS, Dolcourt B (2007). Effective lactate clearance is associated with improved outcome in post-cardiac arrest patients. Resuscitation..

[28] Lin YR, Syue YJ, Lee TH, Chou CC, Chang CF, Li CJ (2018). Impact of different serum potassium levels on postresuscitation heart function and hemodynamics in patients with nontraumatic out-of-hospital cardiac arrest. Bioinorg Chem Appl.

